# The Value of the First Repetition: Force, Impulse, and Linear Velocity in Flywheel Deadlifts and Their Link to Maximal Free-Weight Strength

**DOI:** 10.3390/sports13100345

**Published:** 2025-10-03

**Authors:** Athanasios Tsoukos, Gregory C. Bogdanis

**Affiliations:** School of P.E. and Sport Science, National and Kapodistrian University of Athens, 17237 Athens, Greece; gbogdanis@phed.uoa.gr

**Keywords:** time under tension, moments of inertia, weightlifting, kinetic analysis, kinematic analysis, strength assessment

## Abstract

The purpose of this study was threefold: (a) to analyze differences in mean force, impulse, mean concentric and eccentric velocity, and peak concentric velocity across six repetitions of the flywheel deadlift exercise, with a particular focus on the first repetition initiated from zero momentum; (b) to explore relationships between these kinetic and kinematic variables and one-repetition maximum (1-RM) performance in the free-weight deadlift; (c) to examine the effects of different flywheel inertial loads on the relationships among mean force (MF), impulse, time under tension (TUT), and velocity, with the aim of identifying the most valid and reliable parameter for flywheel load prescription. Thirteen resistance-trained men (24.7 ± 5.0 y; 82.2 ± 11.7 kg; 1-RM deadlift: 174 ± 24 kg) performed six repetitions of the flywheel deadlift against six inertial loads (0.025 to 0.145 kg∙m^2^) on a kBox 5 device. Results showed that although the first repetition had 25–30% lower mean concentric velocity and 7–11% lower mean force compared to subsequent repetitions (*p* < 0.001), it exhibited 4–8% higher impulse due to the 14–20% longer time under tension. MF, velocity, and impulse in the first repetition showed moderate-to-strong correlations with 1-RM (r = 0.58 to 0.85, *p* < 0.05), particularly at the two higher inertia loads. MF plateaued at moderate inertia loads, while impulse and TUT increased linearly with increasing inertial load and demonstrated the strongest and most consistent relationships with inertial load (r = 0.99 ± 0.01 and 0.97 ± 0.02, *p* < 0.001), enabling individualized flywheel training prescription. This study highlights the distinct value of the first repetition in flywheel deadlifts and its practical value for both assessment and training. Also, it suggests that impulse and TUT may be used as simple and practical flywheel exercise prescription variables.

## 1. Introduction

Flywheel-based resistance systems provide a distinctive alternative to gravity-dependent training methods and have been shown to offer several practical and physiological benefits [1,2,3]. These devices are lightweight and portable, present a lower risk of injury, and deliver resistance regardless of movement direction or plane [1,2,4]. Because they do not rely on fixed weights, there are no “sticking points” during the concentric phase, and resistance naturally adapts to the user’s current capacity, even as fatigue develops [1,2,4]. Under certain circumstances, they can also be used to create eccentric overload- a scenario in which the eccentric output (force, power or velocity) surpasses that produced concentrically [1,5]. In contrast to free weights, where the load is determined by mass and gravity, the resistance in a flywheel exercise derived from the rotational inertia of the wheel [6]. This characteristic makes the method viable in environments without gravity, such as spaceflight [3]. During an upward effort—for example, standing from a squat—the rotation of the wheel accumulates kinetic energy [2,6,7,8,9]. When transitioning to the downward phase, this stored energy is fed back into the system, requiring the lower limb muscles to absorb it [2,6,9]. In this phase, the eccentric contraction acts as a braking mechanism to control the motion [10,11,12]. It is worth noting that this eccentric over-loading is not automatically maximized simply by using the device. The actual magnitude depends on the velocity achieved in the concentric movement and on how the user applies braking in the eccentric phase [1,13,14]. To deliberately create high eccentric demands (eccentric overload), the user must delay deceleration and then apply a strong braking effort toward the final portion—roughly the last third—of the lowering movement [1,13,14].

Resistance during flywheel training can be adjusted by selecting flywheels with varying moments of inertia, with higher inertia producing greater resistance [5,15,16,17]. Carroll et al. (2017) investigated the kinetic properties of flywheel squats performed with low moments of inertia and reported that both peak force and net impulse significantly increased with higher inertia loads (Load 1: 0.010 kg∙m^2^, Load 2: 0.025 kg∙m^2^, and Load 3: 0.050 kg∙m^2^) [15]. On the other hand, a recent study showed that mean force exhibited a relatively low coefficient of determination (r^2^ ranging from 0.21 to 0.48) across various fitting models—linear, logarithmic, polynomial and exponential—when examining a broad range of moments of inertia (0.025–0.125 kg∙m^2^), especially when compared to mean velocity, which showed much stronger correlations (r^2^ between 0.88 and 0.97) [18]. Additionally, McErlain-Naylor et al. (2021) recommended using velocity rather than power to prescribe and monitor flywheel squat exercise intensity, as both peak and mean concentric velocities (r^2^ = 0.89 to 0.97 for linear and non-linear models) tended to decrease with increased inertia [19]. These findings highlight velocity as a more reliable parameter than force or power for load management in flywheel training across different inertia settings [18,19,20,21].

When analyzing kinetic, kinematic, or performance variables during flywheel resistance training, researchers often exclude the initial 1–4 repetitions, considering them necessary for accelerating the system and reaching the target velocity [1,13,22]. These early repetitions are commonly referred to as “wasted repetitions” [1,13]. However, the mechanical characteristics of the first repetition—starting from zero momentum—may provide unique and meaningful information about an individual’s maximal strength expression and neuromuscular control. Labeling the initial repetitions as “wasted” may overlook valuable kinetic and kinematic information that could be critical in understanding maximal strength expression and training adaptations. However, in practical scenarios such as weightlifting or sprint starts (block or crouch starts), athletes initiate their efforts from a state of zero momentum [23,24]. The deadlift is a fundamental multi-joint exercise that demands high levels of coordinated force production, which also begins from zero velocity or momentum [23,25,26,27,28], making it an ideal movement to study the relationship between flywheel training variables and maximal strength [29,30,31]. Compared with free-weight deadlifts, flywheel deadlifts provide variable resistance determined by the wheel’s inertia, yielding distinct kinematic and kinetic profiles relative to the constant gravitational load of free weights. Preliminary analyses [32] indicate that, for deadlifts, free weights tend to generate greater peak knee flexion angle and knee extension moments, while hip, lumbar, and ankle impulse moments are often similar or slightly lower under flywheel conditions, particularly during the first repetition where momentum is minimal [32]. Furthermore, during multi-joint, lower-limb exercises such as squats and lunges, flywheel resistance increases the mechanical demands on the hip extensors and ankle plantar flexors while reducing those on the knee extensors [33]. These findings underscore the unique mechanical characteristics of flywheel exercises and provide a rationale for analyzing the first repetition to elucidate maximal strength expression and neuromuscular activation. Therefore, it is important to investigate the characteristics of these initial flywheel repetitions from both kinetic and kinematic perspectives, as well as to explore whether these early parameters correlate with overall performance outcomes such as one-repetition maximum (1-RM) performance in exercises like the deadlift. Investigating these early repetitions could improve load prescription and monitoring strategies, enhancing the effectiveness of flywheel resistance training in both athletic and rehabilitation settings. Therefore, the purpose of the present study was threefold: (a) to analyze the differences across six repetitions, with a particular focus on the first repetition that starts from zero momentum, in mean force, impulse, mean concentric and eccentric linear velocity, as well as peak concentric velocity during flywheel deadlifts; (b) to explore the relationships between these kinetic and kinematic variables and one-repetition maximum (1-RM) performance in the dynamic deadlift; (c) to examine the effects of different inertial loads on the relationships among force, impulse, time under tension, and velocity, in order to identify the most valid and reliable parameter for flywheel load prescription and monitoring, with practical implications for optimizing training intensity and athlete performance.

## 2. Materials and Methods

### 2.1. Participants

Thirteen male participants volunteered for this study (age: 24.7 ± 5.0 years, height: 1.78 ± 0.08 m, body mass: 82.2 ± 11.7 kg, % body fat: 12.3 ± 4.2%, 1-RM free-weight deadlift: 173.7 ± 24.3 kg). They were experienced in weightlifting, Crossfit, and strength and power training, each having a minimum of three years of consistent practice. Although most participants had prior experience with flywheel training devices and had also participated in a previous study on the acute effects of flywheel exercise [25], all completed two familiarization sessions to ensure consistent and proper technique during the flywheel deadlift exercise. Eligibility criteria included the following: (a) no use of nutritional supplements or performance-enhancing drugs, (b) absence of musculoskeletal injuries in the last 12 months, (c) non-smoking status, (d) avoidance of any training sessions for at least 48 h before each laboratory visit, and (e) stable dietary habits 24 h prior to testing. An a priori power analysis was conducted using G*Power (v. 3.1.9.7) to determine the required sample size. The analysis employed a medium effect size (f = 0.25), an alpha level of 0.05, a statistical power of 0.80, and an estimated correlation of 0.7 among repeated measures, resulting in a recommended sample size of 12 participants. Therefore, the sample of 13 participants was sufficient to reliably detect medium-sized effects. Prior to participation, all volunteers were fully informed about the study protocol, potential risks, and their rights, including the freedom to withdraw at any time, without penalty. Written informed consent was obtained from each participant. The research protocol was approved by the Institutional Review Board (Approval no. 1700/18-11-2024) and conducted in accordance with the ethical principles outlined in the Declaration of Helsinki of 1964 as revised by the World Medical Association in 2024.

### 2.2. Research Design

A repeated-measures design was employed. Participants performed one set of six repetitions of flywheel deadlifts using a kBox 5 device (Exxentric, AB TM, Bromma, Sweden) at six different moments of inertia: 0.025, 0.05, 0.075, 0.10, 0.125, and 0.145 kg∙m^2^. These loads were selected based on published guidelines for flywheel training, which recommend low-to-medium loads for power development, medium-to-high loads for strength, and low-to-high loads for injury prevention depending on the exercise and training goal [1]. Additionally, the selected loads were aligned with the available equipment, ensuring the safe and effective execution of the flywheel deadlift. The dependent variables measured for each repetition and inertial load included mean force, impulse, time under tension, mean concentric and eccentric linear velocity, and peak concentric linear velocity. Additionally, the study examined the relationships between these flywheel exercise variables and the participants’ one repetition maximum (1-RM) performance in the traditional free-weight deadlift. Participants completed two familiarization sessions to ensure proper technique, followed by three experimental sessions. During the familiarization sessions, participants performed 2–3 sets of 6–8 repetitions across the range of inertial loads used in the study. Each repetition was closely monitored by the supervising researcher, who provided verbal feedback and corrections on stance, grip, lower back alignment, and movement trajectory. Participants practiced initiating the pull from zero momentum, achieving full hip and knee extension, and applying proper eccentric braking at the end of the downward phase. Technical proficiency was considered adequate when participants consistently performed the movement with correct form, full range of motion, and smooth transition between concentric and eccentric phases across all inertia loads [25]. Only after achieving this standard were participants allowed to proceed to the experimental sessions. Two of the experimental sessions were conducted on the flywheel device, during which the kinetic and kinematic variables were recorded with appropriate instrumentation. The third experimental session involved assessment of the 1-RM deadlift using free-weights. The order of the different moments of inertia, as well as the experimental sessions were randomized and counterbalanced to minimize order effects. Before all familiarization and flywheel experimental sessions, participants performed a standardized warm-up (general and specific), as described in a previous study [25]. For the free-weight 1-RM deadlift assessment, the protocol recommended by the National Strength and Conditioning Association (NSCA) was followed [34].

### 2.3. Flywheel Deadlifts

The flywheel deadlift exercise was executed using a kBox 5 flywheel device (Exxentric, AB TM, Bromma, Sweden). Participants stood barefoot on two force plates placed on top of the flywheel box, maintaining a hip-width stance. The movement started from a low position, with a straight (neutral) lower back and bent knees [25]. They gripped the device’s mini barbell, which was set at mid-shin level. From this position, with elbows kept straight throughout, participants performed a powerful upward pull, drawing the barbell close to the body until reaching a fully upright posture. Additionally, participants were instructed to perform plantar flexion at the ankle, finishing the movement “on their toes” to actively engage the calf muscles [25].

### 2.4. Mean Force, Velocity, Impulse, and Time-Under-Tension During Flywheel Deadlifts

Ground reaction forces were measured for each inertia load and repetition using two dual-axis force plates (PS2142; PASCO Scientific, Roseville, CA, USA) placed on top of the flywheel box under the participants’ feet. The force plates were connected to a computer via an interface (SPARKlink Air; PS-2011; PASCO Scientific, Roseville, CA, USA) and synchronized for data acquisition at a sampling frequency of 1000 Hz. Data were processed using a customized recording template within PASCO Capstone software (version 2.6; PASCO scientific) [35]. Mean force output was calculated by summing the mean forces recorded by the two force plates. Impulse (IMP) was computed as the integral (area) under the vertical ground reaction force-time curve [25,36] and time under tension (TUT) was determined for the entire repetition [25,37,38]. Concentric and eccentric phases were not separated, as the study focused on total mechanical demand per repetition. We also calculated the total IMP and TUT for all six repetitions at each inertia load. Mean concentric velocity (MV), mean eccentric velocity (MECCV), and peak concentric velocity (PV) were measured using a linear position transducer (Tendo Power Analyzer System v. 314, TENDO Sports Machines, Trencin, Slovak Republic), which was connected via Bluetooth to a computer running the corresponding software (Tendo Sports Machine software v. 7.4.1.0) [35,39,40]. The transducer was positioned beneath the flywheel device, with its cable attached to the mini barbel using a strap [16]. The force plates and linear position transducer were not synchronized. Force plates captured only force and time-related variables, while the Tendo system recorded velocity metrics independently.

### 2.5. Free-Weight 1-RM Deadlift Assessment

The participants performed a warm-up protocol consisting of 5 min of light pedaling on a cycle ergometer set at 50–60 W, followed by 5 min of dynamic mobility work. This included walking quadriceps stretches, high knee lifts, lunges with a torso twist, bodyweight squats, and single-led deadlifts, all aimed at engaging the lower limbs and lumbar region. Subsequently, participants completed five repetitions of the “good morning” exercise with a 15 kg barbell, three submaximal bodyweight jump squats, and six pogo jumps [25]. After this they performed 5–6 repetitions at 50% of the predicted 1-RM, 3–4 repetitions at the predicted 75% 1-RM and 3 to 5 sets of one repetition to determine the 1-RM [34,41]. To perform the deadlift exercise, the participants stood barefoot with their feet flat, positioned between hip- and shoulder-width apart, and toes slightly turned outward. They squatted down with the hips lower than the shoulders and grasped the barbell with a closed pronated grip, slightly wider than shoulder-width and outside knees. The back was maintained in a neutral position, with the scapulae retracted, chest up, dead aligned with the spine, heels in contact with the floor, and the bar positioned approximately 1–3 cm in front of the shins. From this position, the bar was lifted by simultaneously extending the hips and knees while keeping the torso angle constant, spine neutral, and bar close to the body. Upon reaching full hip and knee extension, the bar was lowered in a controlled manner by flexing the hips and knees, maintaining a neutral spine until it returned to the floor. On some occasions, the participants allowed the bar to drop from their hands during the final phase of the descent.

### 2.6. Main Trials on the Flywheel Device

The two main trials on the flywheel device and the free-weight 1-RM deadlift assessment were performed in a randomized, counterbalanced order, with a 5–7-day interval between sessions. In each session, participants completed the same standardized warm-up protocol as described above [24]. Five minutes after the warm-up, they performed a familiarization set on the flywheel device (1 set of 6 repetitions) using a medium inertia of 0.05 kg∙m^2^ at submaximal effort. Following a 3 min rest, they completed another submaximal set of 5 repetitions at the predetermined inertia. For the main trials, participants performed three maximal-effort sets of six repetitions at different inertia loads. On a given day, three of the six inertial loads (0.025, 0.05, and 0.075 kg∙m^2^, for example) were tested, while the remaining three inertia load (0.1, 0.125, and 0.145 kg∙m^2^) were completed in a second session 5–7 days later. The order of inertia loads was randomized and counterbalanced, with 5 min of rest between sets.

### 2.7. Statistical Analysis

All variables—mean velocity (MV), peak velocity (PV), eccentric mean velocity (MECCV), impulse (IMP), time under tension (TUT), and mean force (MF)—are reported as means ± standard deviations. Statistical analyses were performed using IBM SPSS Statistics (version 28.0; IBM Corp., Armonk, NY, USA). A two-way repeated-measures ANOVA was conducted to examine differences between variables across the six inertial loads and six repetitions, with Tukey post hoc tests applied where appropriate. To compare the strength of the inertia–force, inertia–impulse, inertia–time under tension, and inertia–velocity relationships, and to identify which parameter provides the most valid and reliable indicator for flywheel load prescription and monitoring, these relationships were modeled using linear regression. Τhe average of repetitions 1–6 was used for this purpose. For pairwise comparisons, effect sizes were calculated using Hedges’ g, and interpreted as small (<0.3), medium (0.3–0.8), or large (>0.8). Pearson’s correlation coefficients were calculated to assess relationships between variables, and the correlation coefficients were transformed into Fisher’s Z scores to enable comparison across conditions. A one-way repeated-measures ANOVA on Fisher’s Z-transformed values was used to evaluate differences in correlation strength across conditions. Statistical significance was accepted at *p* < 0.05.

## 3. Results

### 3.1. Mean and Peak Concentric Velocity

**Figure 1:**
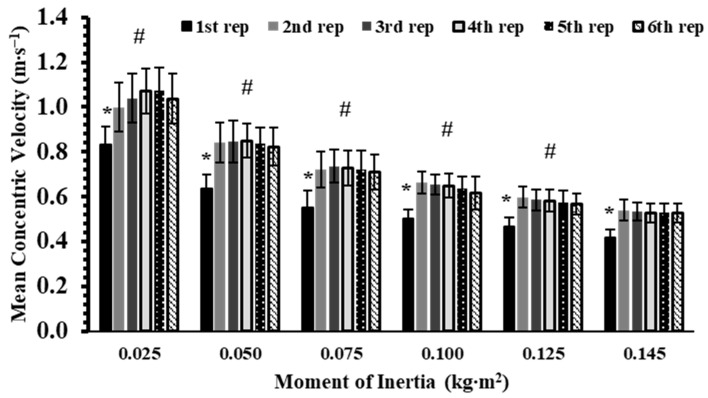
Mean velocity (MV) across six repetitions at six different inertial loads (0.025–0.145 kg∙m^2^). *: *p* < 0.01, 1st repetition vs. all other repetitions at the same inertial load; #: *p* < 0.01, vs. all repetitions at all higher inertial loads.

For mean concentric velocity a significant interaction effect was found (*p* < 0.001, η^2^p = 0.42). Tukey’s post hoc tests showed that, within each inertia load, the first repetition had significantly lower MV than the subsequent five repetitions by about 25–30% (Hedges’ g: from 1.67 to 3.44; *p* < 0.001; Figure 1). Between-inertia comparisons indicated that MV decreased progressively with increasing inertia (Hedges’ g: from 1.52 to 6.40; *p* < 0.05)

**Figure 2:**
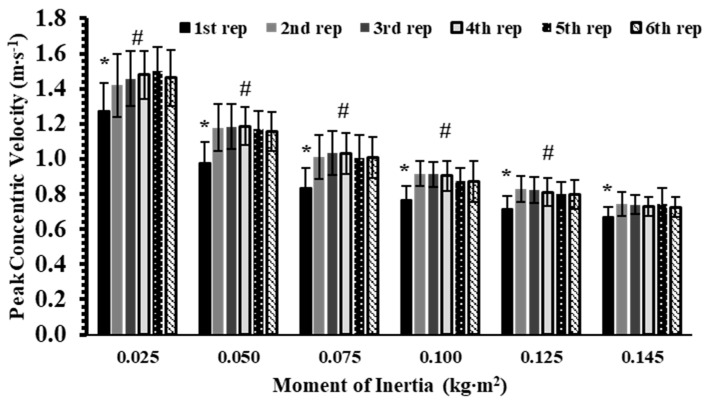
Peak velocity (PV) across six repetitions at six inertial loads (0.025–0.145 kg∙m^2^). *: *p* < 0.01, 1st repetition vs. all other repetitions at the same inertial load; #: *p* < 0.01, vs. all repetitions at all higher inertial loads.

The analysis revealed a significant interaction effect for peak concentric velocity (*p* < 0.001, η^2^p = 0.33). Tukey’s post hoc tests showed that, within each inertia load, the first repetition had significantly lower PV than the subsequent five repetitions by about 10–22% (Hedges’ g: from 0.94 to 1.62; *p* < 0.001; Figure 2). Between-inertia comparisons indicated that PV decreased progressively with increasing inertia (Hedges’ g: from 1.48 to 6.30; *p* < 0.05).

### 3.2. Mean Eccentric Velocity

**Figure 3:**
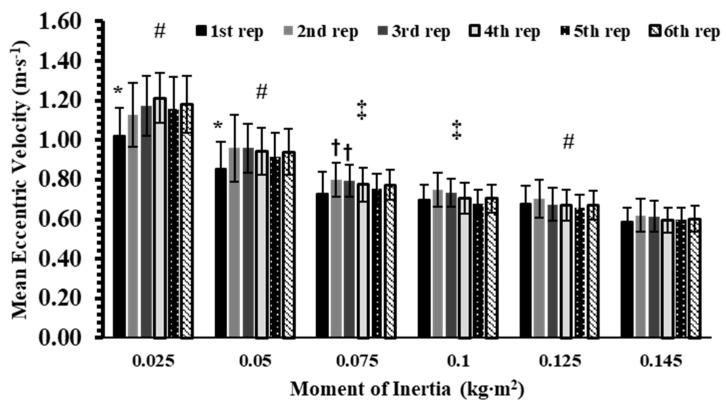
Mean Eccentric velocity (MECCV) across 6 repetitions at 6 inertial loads (0.025–0.145 kg∙m^2^). *: *p* < 0.05, 1st repetition vs. all other repetitions at the same inertial load; #: *p* < 0.01, vs. all repetitions at all higher inertial loads; †: *p* < 0.01 vs. the 1st repetition at the same inertia load; ‡: *p* < 0.05 vs. all other repetitions at higher inertial loads except the immediately higher inertial load (0.075 vs. 0.10 and 0.10 vs. 0.125 kg∙m^2^).

The analysis showed a significant interaction effect for mean eccentric velocity (*p* < 0.001, η^2^p = 0.37). Post hoc analyses showed that, within each inertia, the 1st repetition had significantly lower MECCV than repetitions 2–6 at 0.025 and 0.05 kg∙m^2^ (Hedges’ g: from 0.46 to 1.36; *p* < 0.05), and lower than the 2nd and 3rd repetitions at 0.075 kg∙m^2^ (Hedges’ g: from 0.64 to 0.69; *p* < 0.05, Figure 3). No other within-inertia differences were observed. Between inertias, MECCV at 0.025 and 0.05 kg∙m^2^ was significantly lower than at all higher inertias across every repetition (Hedges’ g: from 0.99 to 5.99; *p* < 0.01). In contrast, 0.075 kg∙m^2^ produced higher MECCV than 0.125 and 0.145 kg∙m^2^ (Hedges’ g: from 0.50 to 2.17; *p* < 0.05) but did not differ from 0.10 kg∙m^2^. Similarly, 0.10 kg∙m^2^ was higher than 0.145 kg∙m^2^ (Hedges’ g: from 1.12 to 1.46; *p* < 0.05), but showed no difference compared with 0.125 kg∙m^2^.

### 3.3. Impulse and Time Under Tension

**Figure 4:**
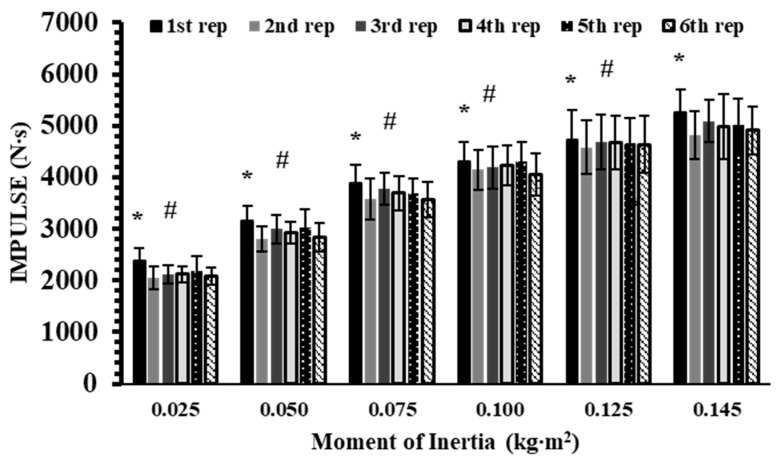
Impulse (IMP) across six repetitions at six inertial loads (0.025–0.145 kg∙m^2^). *: *p* < 0.01, 1st repetition vs. all other repetitions at the same inertial load; #: *p* < 0.01, vs. all repetitions at all higher inertial loads.

The analysis did not reveal a significant interaction effect for IMP (*p* = 0.34, η^2^p = 0.08). However, significant main effects were observed for inertia (*p* < 0.001, η^2^p = 0.98) and repetition (*p* < 0.001, η^2^p = 0.65). Post hoc analyses showed that IMP increased progressively with higher inertia loads (Hedges’ g: from 0.68 to 7.18; *p* < 0.01). Across repetitions, the 1st repetition produced 4–8% greater IMP than all subsequent repetitions within each inertial load (Hedges’ g: from 0.13 to 0.25; *p* < 0.01). (*p* < 0.05; Figure 4).

**Figure 5:**
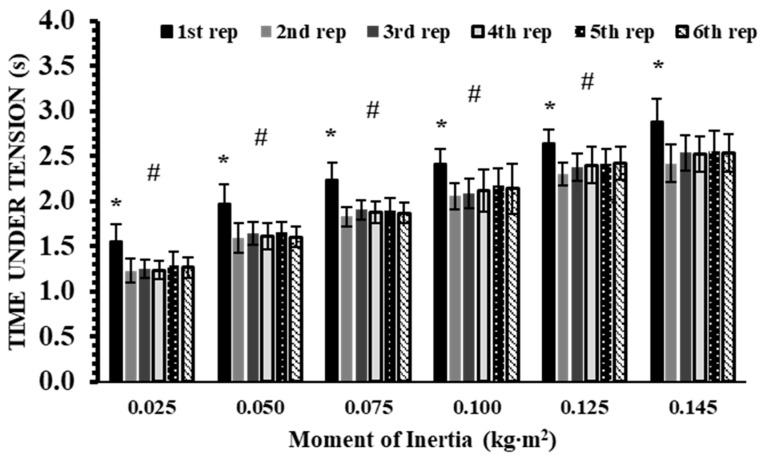
Time under tension (TUT) across six repetitions at six inertial loads (0.025–0.145 kg∙m^2^). *: *p* < 0.01, 1st repetition vs. all other repetitions at the same inertial load; #: *p* < 0.01, vs. all repetitions at all higher inertial loads.

No significant interaction effect was found for TUT (*p* = 0.29, η^2^p = 0.09). However, significant main effects were observed for inertia (*p* < 0.001, η^2^p = 0.96) and repetition (*p* < 0.001, η^2^p = 0.87). Post hoc analyses showed that TUT increased progressively with higher inertia loads (Hedges’ g: from 0.65 to 5.67; *p* < 0.01, Figure 5)). Across repetitions, the 1st repetition produced 14–20% greater TUT than all subsequent repetitions (Hedges’ g: from 0.58 to 0.80; *p* < 0.01).

Total IMP and TUT across inertial loads are presented in Table 1. Both IMP and TUT progressively increased with increasing inertial load.

### 3.4. Mean Force (MF)

**Figure 6:**
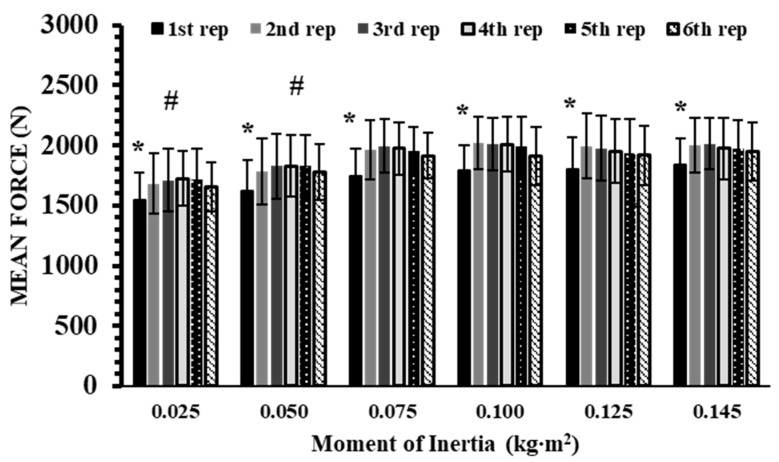
Mean Force (MF) across six repetitions at six inertial loads (0.025–0.145 kg∙m^2^). *: *p* < 0.01 vs. all other repetitions within the same inertial load; #: *p* < 0.01 vs. all corresponding repetitions at higher inertial loads.

A significant interaction effect was found for mean force (*p* < 0.001, η^2^p = 0.17). Post hoc analyses showed that, within each inertia load, the first repetition had 7–11% lower MF than the subsequent five repetitions (Hedges’ g: from 0.45 to 1.07; *p* < 0.001; Figure 6). Between-inertia comparisons indicated that 0.025 kg∙m^2^ differed from all higher inertias across every repetition (Hedges’ g: from 0.28 to 1.40; *p* < 0.01), and 0.05 kg∙m^2^ also differed from all higher inertias (Hedges’ g: from 0.50 to 0.93; *p* < 0.01). No other differences were observed.

### 3.5. Correlations Between Velocity Metrics and 1-RM Across Inertia Levels

Correlations between velocity variables (mean concentric, peak concentric, and mean eccentric) and free-weight 1-RM deadlift ranged from low to very high depending on inertia and repetition (Appendix A Table A1 and Table A2). In general, the first repetition consistently produced the strongest associations with 1-RM, particularly for mean and peak concentric velocity at low (0.025 kg∙m^2^) and moderate-to-high inertias (0.100–0.145 kg∙m^2^), where correlations were high to very high. Averaging repetitions (Rep 1–6) yielded the highest overall correlations; however, the first repetition alone provided comparable predictive validity, highlighting its practical value for estimating maximal strength. Detailed correlation coefficients are presented in Appendix A.

### 3.6. Correlations Between Impulse and 1-RM Across Inertia Loads and Repetitions

Significant correlations between IMP and deadlift 1-RM were observed across several inertia loads, with the first repetition showing moderate to strong associations at 0.025 (r = 0.62, *p* = 0.023), 0.05 (r = 0.62, *p* = 0.024), 0.100 (r = 0.57, *p* = 0.042), 0.125 (r = 0.79, *p* = 0.001), and 0.145 kg∙m^2^ (r = 0.58, *p* = 0.039) (Appendix A Table A3). Subsequent repetitions generally maintained or strengthened these relationships, particularly at 0.075–0.125 kg∙m^2^. and total IMP also demonstrated meaningful associations with 1-RM at moderate-to-high inertias (r = 0.63–0.67, *p* ≤ 0.021), highlighting both single- and cumulative-repetition predictive value.

### 3.7. Correlations Between Mean Force and 1-RM Across Inertia Loads and Repetitions

Significant correlations between mean force and 1-RM were observed primarily at moderate-to-high inertia loads (Appendix A Table A4). At 0.05 kg∙m^2^, Rep 1 showed a significant correlation (r = 0.57, *p* = 0.042), while correlations at 0.025 kg∙m^2^ were not significant. Strong and consistent significant correlations were detected at 0.075–0.145 kg∙m^2^ across nearly all repetitions (r = 0.76–0.92, *p* ≤ 0.006), as well as for averaged repetitions (Rep 1–6 and Rep 2–6, r = 0.77–0.90, *p* ≤ 0.002). These findings indicate that mean force is a robust predictor of 1-RM at moderate-to-high inertia, with both single repetitions and averaged values providing highly reliable associations.

### 3.8. Inertial Load vs. Response Relationships: Slopes, Correlations, and Fisher’s Z for Flywheel Deadlift Variables

Table 2 shows the inertial load vs. response relationships for flywheel deadlift variables across repetitions 1–6. The highest Fisher’s Z-transformed values were observed for IMP and TUT, which were significantly greater than all other variables (*p* < 0.01). MV and PV also showed significantly higher Fisher’s Z values compared to MECCV and MF (*p* < 0.01), while MECCV was significantly greater than MF (*p* < 0.01). These results indicate that IMP and TUT exhibited the strongest inertia–response relationships among all variables, followed by MV and PV, with MECCV and MF showing progressively weaker relationships.

## 4. Discussion

The purpose of the present study was threefold: (a) to analyze the differences in kinematic and kinetic variables across six flywheel deadlift repetitions and six inertial loads, with a particular focus on the first repetition that starts from zero momentum; (b) to explore the relationships between these kinetic and kinematic variables and one-repetition maximum (1-RM) performance in the dynamic deadlift; (c) to analyze and compare the effects of different inertia loads on the relationships between force, IMP, TUT, and velocity, in order to identify which parameter provides the most valid and reliable indicator for flywheel load prescription and monitoring. The study revealed that the first repetition consistently differs from the subsequent ones across all inertial loads, showing lower mean and peak concentric velocity, mean force, and eccentric velocity, but higher TUT and IMP. Despite these differences, the first repetition also demonstrated strong and consistent associations with 1-RM deadlift performance, particularly at both very low and high inertias. Between-inertia comparisons showed that increases in inertia were accompanied by reductions in concentric and eccentric velocities, while impulse and time under tension increased progressively, whereas MF rose up to 0.075 kg·m^2^ before reaching a plateau, confirming the expected load–velocity relationship in flywheel deadlifts. It is worth noting that the inertia at which MF plateaued varied slightly between participants, reflecting individual differences in strength, neuromuscular capacity, and ability to generate force at higher loads. Anthropometric factors, such as limb lengths, body mass, and muscle cross-sectional area, may also influence individual force–velocity profiles and the point at which mean force plateaus. This suggests that while 0.075 kg·m^2^ represents the average plateau point in our sample, coaches may need to adjust flywheel loads individually to account for athlete-specific force capacities. When comparing inertial load vs. outcome relationships, Fisher’s *z* values indicated that IMP and TUT were the most valid and reliable indicators for flywheel load prescription and monitoring, outperforming velocity- and force-based metrics. In practical terms, this means that coaches and practitioners can rely more confidently on IMP and TUT when adjusting inertial loads, as these variables provide the most stable and sensitive feedback across different loading conditions. By contrast, force- and velocity-based measures may offer supplementary information but appear less consistent for monitoring purposes. These findings emphasize the distinct role of the first repetition and underscore the superior utility of IMP and TUT for strength assessment in flywheel training, provided that participants initiate the movement with maximal effort from the correct starting position.

The first repetition in flywheel resistance training is mechanically and physiologically distinct from subsequent repetitions. It begins with a state of zero momentum, requiring higher neuromuscular activation to initiate the movement, and involves unique acceleration and deceleration phases. Traditionally, researchers have often excluded the initial 1–4 repetitions, considering them necessary only to accelerate the flywheel and reaching the target velocity [1,13,22]. Suchomel et al. (2019) even referred to these as “wasted repetitions” [1,13] suggesting that they do not contribute fully to the intended stimulus [1,13]. However, the present study demonstrates that when participants start from a low initial position with maximum effort, the first repetition—despite having lower velocities—produces a 4–8% higher force impulse than subsequent repetitions, due to its longer TUT, even though it exhibits slightly lower MF. This finding challenges the conventional view of early repetitions as “wasted,” highlighting that the first repetition provides a meaningful and functionally important neuromuscular stimulus. Previous research has emphasized the importance of IMP and TUT in driving training adaptations [25,36,42,43]. Furthermore, in practical sports contexts such as weightlifting, powerlifting, and track and field starts, athletes often initiate efforts from zero momentum [23,24,44]. Therefore, the first repetition during flywheel training is functionally important, and strength and conditioning coaches should deliberately monitor and train this initial effort. In addition to practical implications, flywheel training with maximal intent from the first repetition provides important physiological benefits. As highlighted by Behm et al. (2024), explosive or ballistic contraction intent enhances motor unit recruitment and firing rates, improving neuromuscular activation and motor control [45]. These adaptations suggest that starting repetitions with maximal effort against an individualized inertial load that maximizes IMP and TUT not only provides a valid mechanical stimulus but also promotes meaningful neuromuscular improvements.

The present study demonstrated an inverse relationship between inertial loads and mean concentric velocity, peak concentric velocity, and mean eccentric velocity. Similar trends have been reported by Martín-Rivera et al. (2022) and Zhu et al. (2025), who examined flywheel squats across a wide range of intensities using linear encoders and found slightly lower coefficients of determination between concentric velocity and levels of inertial load (r^2^ = 0.80 and 0.77, respectively) compared with the present study (Table 2) [20,21]. These small discrepancies may be explained by differences in exercise selection (squat vs. deadlift) and encoder placement. Additional factors that may contribute to the modest discrepancies in correlation coefficients observed between studies include differences in participant characteristics (e.g., training experience, strength levels, and sex), as well as variations in data collection protocols and familiarization procedures. Importantly, the present findings revealed that mean eccentric velocity yielded significantly lower Ficher’s Z transformed values of Pearson’s R compared with concentric velocities (Table 2). This finding suggests that concentric velocities should be prioritized when prescribing or monitoring flywheel training loads, as they track changes in inertia more reliably. In contrast, mean eccentric velocities may be less useful for practical load adjustments due to the weaker and variable associations. Comparable results were observed by McErlain-Naylor et al. (2021), who recommended concentric velocity over power for prescribing and monitoring flywheel squat exercise intensity, as both peak and mean concentric velocities declined consistently with increasing inertia, whereas mean eccentric velocity demonstrated weaker associations (r^2^ = 0.73 vs. 0.98 and 0.95, respectively) [19]. Collectively, these findings suggest that concentric velocities provide a more valid and reliable indicator for prescribing intensity in flywheel resistance training, whereas eccentric velocity appears less suitable.

This is the first study to demonstrate that IMP and TUT show stronger linear relationships with inertia loads compared to mean force. The relatively weaker relationship observed for mean force may be explained by the fact that force values plateaued at inertias above 0.075 kg∙m^2^ (Figure 6). Mechanically, the torque required to accelerate or decelerate the flywheel is proportional to its moment of inertia (*T* = *I*·*α*), while the corresponding linear force is given by *F* = *T*/*r* [9]. Since the shaft radius was identical across all inertias, increases in inertial load (i.e., moment of inertia of the disk) required proportionally greater torque. However, the user’s capacity to generate higher force reached a ceiling beyond 0.075 kg∙m^2^ in our sample. Consequently, mean force leveled off, while reductions in acceleration produced progressively slower velocities and longer TUT, reinforcing the strong linearity of both IMP and TUT with inertia. Because IMP is the product of force and time, and force alone did not yield a strong correlation, TUT appears to be the main factor driving the robust linearity of IMP. This finding suggests that TUT may represent the simplest and most reliable parameter for prescribing intensity during flywheel resistance training. Importantly, TUT can be assessed with minimal equipment, even with a standard stopwatch, making it highly practical for coaches and practitioners. However, when using a stopwatch to measure TUT, coaches should consider potential variability due to human reaction time. Standardizing measurement procedures and practice over time may further improve accuracy in practical settings. Beyond its accessibility, TUT also aligns with mechanistic perspectives: prolonged muscle activation and loading duration enhance neuromuscular adaptations, facilitate energy transfer, and increase metabolic stress [15,35,38,46,47]. Moreover, the consistency of IMP and TUT across different inertias underscores their potential as universal indicators for monitoring and individualizing flywheel training loads. Collectively, these findings highlight TUT as both a practical and physiologically meaningful tool for load prescription in flywheel resistance training. However, it should be noted that these findings are based on experienced male athletes, and the strength of these relationships may differ in other populations, such as untrained individuals, female athletes, or athletes with lower strength levels. Future studies should examine whether IMP and TUT maintain the same linearity and reliability across different training backgrounds and population groups. These findings have practical implications for training periodization. By monitoring IMP and TUT across different inertial loads, coaches can individualize load prescription and progression, adjusting intensity and volume according to athlete-specific responses. Deliberate maximal-effort first repetitions may be strategically incorporated to enhance neuromuscular activation, while monitoring the individual load–velocity and force–time profiles can guide exercise sequencing and progression across strength and power phases. Integrating these principles may help optimize training adaptations in both short- and long-term programs.

The present study also found strong correlations between kinematic and kinetic parameters during flywheel resistance training and free-weight 1-RM deadlift performance. A recent study examined the relationship between free-weight and flywheel loading patterns, showing that while free-weight squats produce higher relative speed and power at matched workloads, flywheel exercises elicit greater vertical forces at moderate-to-high resistances [18]. These findings support the notion that, despite differences in movement mechanics, flywheel deadlifts can provide comparable or even greater mechanical stimulus relative to free-weight 1-RM performance, particularly when effort is maximal. Consequently, the strong correlations observed in the present study between flywheel variables and free-weight 1-RM align with the concept that both modalities share meaningful force–velocity characteristics. A previous study that examined the relationship between bodyweight, absolute and relative strength, and power variables in the flywheel Romanian deadlift, reported large positive correlations between 1-RM and peak concentric power, and between relative strength and both concentric and eccentric peak power, although these associations did not reach statistical significance (*p* > 0.05) [31]. Compared to those findings, the stronger and significant associations observed in the present study may be attributed to differences in exercise selection (deadlift vs. Romanian deadlift), sample characteristics, or the inclusion of additional kinetic and kinematic variables. The sample in that study consisted of ten physically active men [31], whereas the present study included experienced weightlifters, CrossFit athletes, and strength-trained practitioners with extensive resistance training backgrounds. Furthermore, recent meta-analysis has shown that mean and peak velocity measured with inertial devices demonstrate nearly perfect correlations with criterion measures, confirming their validity for exercises such as the bilateral stiff-leg deadlift, while correlations for other power variables (e.g., hip extension, knee bridge) appear weaker [48]. These findings reinforce that exercise specificity and the choice of outcome variables strongly influence the strength of the observed associations between flywheel performance and traditional strength measures.

Several limitations should be acknowledged in the present study. First, although the order of different moments of inertia was randomized and counterbalanced, potential carry-over effects between repetitions with different loads within the same session cannot be completely ruled out. Second, the sample consisted of experienced male athletes, which may limit the generalizability of the findings to other populations, such as female athletes, untrained individuals, or athletes with different training backgrounds. Third, the study focused solely on the flywheel deadlift, and the relationships observed may differ for other exercises or movement patterns commonly used in flywheel training. Fourth, despite careful familiarization, inter-individual differences in technique and adaptation to flywheel devices may have influenced the results. Finally, technical challenges inherent to flywheel training, such as maintaining correct posture, ensuring maximal effort, and controlling velocity, may affect real-world application and should be considered when implementing these findings in practice.

## 5. Conclusions

In summary, this study highlights the distinct value of the first repetition in flywheel deadlifts and its practical value for both assessment and training. Despite slightly lower concentric and eccentric velocities and MF compared to subsequent repetitions, the first repetition consistently produced greater IMP and TUT while showing strong associations with free-weight 1-RM deadlift performance, particularly at high inertia loads. Furthermore, while mean and peak velocity followed the expected inverse relationship with inertia, and mean force plateaued at moderate loads, IMP and TUT increased linearly with increasing inertial load and demonstrated the strongest and most consistent relationships with inertial load, enabling individualized flywheel training prescription. These findings suggest that IMP and TUT may serve as superior metrics for load prescription and monitoring in flywheel resistance training, and that coaches should recognize the functional and adaptive significance of the first repetition, especially in sports where movements often begin from zero momentum. For training implementation, we recommend that practitioners prioritize IMP and TUT as primary metrics for load adjustments. Also, flywheel deadlift training should be executed with maximal effort from the first repetition, while the inertial load should be individualized based on maximum IMP.

## Figures and Tables

**Table 1:** Total impulse (IMP) and total time under tension (TUT) across six inertia loads for repetitions 1–6 and 2–6.

Inertial Load (kg∙m^2^)	Total IMP (N∙s) (1–6 Reps)	TUT (s) (1–6 Reps)
0.025	11,184 ± 4027 *	7.82 ± 0.74 *
0.050	17,744 ± 1419 *	10.09 ± 0.75 *
0.075	22,198 ± 1832 *	11.62 ± 0.65 *
0.100	25,222 ± 2102 *	12.99 ± 0.99 *
0.125	27,962 ± 3000 *	14.57 ± 0.84 *
0.145	30,080 ± 2617	15.47 ± 1.12

*: *p* < 0.01 compared with all higher inertias.

**Table 2:** Inertial load–response relationships (slopes, Pearson’s r, coefficient of determination [R^2^], and Fisher’s Z-transformed values) for flywheel deadlift variables across repetitions 1–6. Data are presented as mean ± SD. MV = mean velocity; PV = peak velocity; MECCV = eccentric velocity; IMP = impulse; TUT = time under tension; MF = mean force. *: IMP and TUT exhibit higher correlations than all other variables (*p* < 0.01), #: *p* < 0.01 MV and PV exhibit higher correlations than MECCV and MF (*p* < 0.01), †: MECCV exhibit higher correlations than MF (*p* < 0.05).

Variable	Statistic	Mean Reps 1–6
MV	SLOPE	−3.88 ± 0.64
	PEARSON r	−0.96 ± 0.03
	R^2^	0.91 ± 0.05
	Pearson’s r (Fisher’s Z-Transformed)	−1.98 ± 0.30 #
PV	SLOPE	−5.54 ± 0.98
	PEARSON r	−0.95 ± 0.03
	R^2^	0.91 ± 0.06
	Pearson’s r (Fisher’s Z-Transformed)	−1.97 ± 0.33 #
MECCV	SLOPE	−4.18 ± 0.96
	PEARSON r	−0.94 ± 0.05
	R^2^	0.88 ± 0.09
	Pearson’s r (Fisher’s Z-Transformed)	−1.85 ± 0.44 †
IMP	SLOPE	23477 ± 2678
	PEARSON r	0.99 ± 0.01
	R^2^	0.97 ± 0.02
	Pearson’s r (Fisher’s Z-Transformed)	2.55 ± 0.33 *
TUT	SLOPE	10.4 ± 1.6
	PEARSON r	0.98 ± 0.01
	R^2^	0.97 ± 0.02
	Pearson’s r (Fisher’s Z-Transformed)	2.59 ± 0.47 *
MF	SLOPE	2266 ± 1388
	PEARSON r	0.78 ± 0.14
	R^2^	0.62 ± 0.21
	Pearson’s r (Fisher’s Z-Transformed)	1.15 ± 0.43

## Data Availability

The full datasets generated and analyzed during the current study cannot be made openly available due to ethical restrictions and participant privacy concerns. The data contain information that could potentially identify individual participants and are available from the corresponding author upon reasonable request.

## Appendix A

**Table A1:** Correlations Between Mean Concentric Velocity and 1-RM Across Inertia loads and Repetitions.

Inertial Load (kg∙m^2^)	Rep 1	Rep 2	Rep 3	Rep 4	Rep 5	Rep 6	Rep 1–6
0.025	0.76	0.56	0.42	0.64	0.69	0.65	0.69
	*p* = 0.003	*p* = 0.045	*p* = 0.154	*p* = 0.018	*p* = 0.009	*p* = 0.016	*p* = 0.010
0.050	0.35	0.27	0.49	0.59	0.48	0.59	0.50
	*p* = 0.247	*p* = 0.374	*p* = 0.090	*p* = 0.035	*p* = 0.098	*p* = 0.033	*p* = 0.081
0.075	0.2647	0.3609	0.3024	0.2268	0.3585	0.4322	0.3431
	*p* = 0.382	*p* = 0.226	*p* = 0.315	*p* = 0.456	*p* = 0.229	*p* = 0.140	*p* = 0.251
0.100	0.373	0.56	0.71	0.70	0.77	0.64	0.73
	*p* = 0.209	*p* = 0.045	*p* = 0.006	*p* = 0.008	*p* = 0.002	*p* = 0.019	*p* = 0.004
0.125	0.80	0.69	0.81	0.82	0.83	0.81	0.83
	*p* = 0.001	*p* = 0.009	*p* = 0.001	*p* = 0.001	*p* = 0.000	*p* = 0.001	*p* = 0.000
0.145	0.71	0.71	0.66	0.67	0.73	0.77	0.79
	*p* = 0.006	*p* = 0.006	*p* = 0.015	*p* = 0.013	*p* = 0.004	*p* = 0.002	*p* = 0.001

**Table A2:** Correlations Between Peak Concentric Velocity and 1-RM Across Inertia loads and Repetitions.

Inertial Load (kg∙m^2^)	Rep 1	Rep 2	Rep 3	Rep 4	Rep 5	Rep 6	Rep 1–6
0.025	0.67	0.51	0.43	0.54	0.52	0.59	0.60
	*p* = 0.012	*p* = 0.075	*p* = 0.142	*p* = 0.054	*p* = 0.066	*p* = 0.035	*p* = 0.029
0.050	0.40	0.21	0.45	0.45	0.34	0.48	0.41
	*p* = 0.171	*p* = 0.484	*p* = 0.121	*p* = 0.123	*p* = 0.250	*p* = 0.098	*p* = 0.160
0.075	0.2865	0.3372	0.3937	0.2086	0.3292	0.3646	0.3315
	*p* = 0.343	*p* = 0.260	*p* = 0.183	*p* = 0.494	*p* = 0.272	*p* = 0.221	*p* = 0.269
0.100	0.61	0.66	0.69	0.55	0.69	0.37	0.63
	*p* = 0.028	*p* = 0.015	*p* = 0.010	*p* = 0.050	*p* = 0.009	*p* = 0.216	*p* = 0.022
0.125	0.73	0.74	0.81	0.85	0.74	0.83	0.82
	*p* = 0.004	*p* = 0.004	*p* = 0.001	*p* = 0.000	*p* = 0.004	*p* = 0.000	*p* = 0.001
0.145	0.85	0.72	0.58	0.70	0.74	0.77	0.77
	*p* < 0.001	*p* = 0.006	*p* = 0.040	*p* = 0.008	*p* = 0.004	*p* = 0.002	*p* = 0.002

**Table A3:** Correlations Between Impulse and 1-RM Across Inertia loads and Repetitions.

Inertial Load (kg∙m^2^)	Rep 1	Rep 2	Rep 3	Rep 4	Rep 5	Rep 6	Rep 1–6	Total Impulse
0.025	0.62	0.79	0.37	0.50	0.65	0.21	0.63	0.00
	*p* = 0.023	*p* = 0.001	*p* = 0.217	*p* = 0.081	*p* = 0.015	*p* = 0.490	*p* = 0.022	*p* = 0.997
0.050	0.62	0.42	0.42	0.39	0.17	0.45	0.46	0.46
	*p* = 0.024	*p* = 0.153	*p* = 0.151	*p* = 0.186	*p* = 0.583	*p* = 0.123	*p* = 0.110	*p* = 0.110
0.075	0.49	0.60	0.66	0.58	0.49	0.60	0.63	0.63
	*p* = 0.087	*p* = 0.031	*p* = 0.015	*p* = 0.039	*p* = 0.088	*p* = 0.030	*p* = 0.021	*p* = 0.021
0.100	0.57	0.56	0.59	0.58	0.57	0.72	0.67	0.67
	*p* = 0.042	*p* = 0.048	*p* = 0.034	*p* = 0.039	*p* = 0.044	*p* = 0.006	*p* = 0.012	*p* = 0.012
0.125	0.79	0.66	0.64	0.46	0.62	0.53	0.66	0.66
	*p* = 0.001	*p* = 0.014	*p* = 0.019	*p* = 0.109	*p* = 0.024	*p* = 0.061	*p* = 0.014	*p* = 0.014
0.145	0.58	0.30	0.39	0.56	0.38	0.58	0.52	0.52
	*p* = 0.039	*p* = 0.313	*p* = 0.182	*p* = 0.046	*p* = 0.201	*p* = 0.039	*p* = 0.068	*p* = 0.068

**Table A4:** Correlations Between Mean Force and 1-RM Across Inertia loads and Repetitions.

Inertial Load (kg∙m^2^)	Rep 1	Rep 2	Rep 3	Rep 4	Rep 5	Rep 6	Rep 1–6
0.025	0.52	0.45	0.30	0.40	0.44	0.30	0.41
	*p* = 0.067	*p* = 0.126	*p* = 0.322	*p* = 0.173	*p* = 0.128	*p* = 0.325	*p* = 0.165
0.050	0.57	0.52	0.54	0.52	0.46	0.41	0.51
	*p* = 0.042	*p* = 0.067	*p* = 0.057	*p* = 0.068	*p* = 0.114	*p* = 0.164	*p* = 0.073
0.075	0.77	0.84	0.90	0.90	0.90	0.89	0.88
	*p* = 0.002	*p* = 0.000	*p* = 0.000	*p* = 0.000	*p* = 0.000	*p* = 0.000	*p* = 0.000
0.100	0.79	0.92	0.89	0.87	0.86	0.89	0.90
	*p* = 0.001	*p* = 0.000	*p* = 0.000	*p* = 0.000	*p* = 0.000	*p* = 0.000	*p* = 0.000
0.125	0.83	0.76	0.79	0.72	0.78	0.74	0.78
	*p* = 0.000	*p* = 0.002	*p* = 0.001	*p* = 0.006	*p* = 0.002	*p* = 0.004	*p* = 0.002
0.145	0.79	0.82	0.83	0.82	0.82	0.84	0.83
	*p* = 0.001	*p* = 0.001	*p* = 0.000	*p* = 0.001	*p* = 0.001	*p* = 0.000	*p* = 0.000
